# Deoxynivalenol and pigs: review of harmful effect of Mycotoxin on swine health

**DOI:** 10.1186/s40813-025-00441-w

**Published:** 2025-05-10

**Authors:** Izabela Malczak, Anna Gajda, Piotr Jedziniak

**Affiliations:** Department of Pharmacology and Toxicology, National Veterinary Research Institute, Partyzantów 57, Pulawy, 24-100 Poland

**Keywords:** Mycotoxins, Deoxynivalenol, Pigs, Toxicity, Feedstuff

## Abstract

Mycotoxins are compounds produced by certain types of fungi, and the mycotoxin one of the most most frequently found in the tested cereal samples is deoxynivalenol (DON), naturally-occurring mycotoxin produced by *Fusarium spp.* An animal sensitive to the effects of this mycotoxin is the pig due to the diet containing primarily cereals and the structure of a digestive system, which causes DON to be quickly absorbed unchanged into the bloodstream and partially metabolized in the liver. An important aspect when considering the toxicity of DON is the occurrence of its modified forms, which can be transformed into DON in the digestive system. The toxic effect of DON can also be caused by other mycotoxins which co-occur in cereals. The toxic effect of DON on the body of pigs was observed mainly in the digestive, immune, and reproductive systems. The noticeable of DON’s toxic effects depends on the exposure time, route of administration and mycotoxin concentration. The changes are mainly caused by impaired gene expression, inhibiting protein synthesis or the effect of DON on pathways in pigs’ bodies. The negative impact of DON on the health of pigs may lead to reduced weight gain, poor health, and increased susceptibility to infections and reproductive disorders. There have also been many methods of DON elimination from cereals, but their efficiency is insufficient.

## Background

Mycotoxins are toxic secondary metabolites produced by certain types of fungi. Thus, mould contamination does not mean the crops must be contaminated with mycotoxins. Fungi capable of producing mycotoxins are also known as toxigenic fungi, and three fungal genera dominate mycotoxin production: *Aspergillus*,* Fusarium*, and *Penicillium* [1]. Mycotoxins enter the food chain through toxic fungi that infect animal or human food before and after harvest, and they can be found in various commodities such as cereals, dried fruits, nuts or spices. Temperature and air humidity are important factors in the production of mycotoxins. Therefore, the occurrence of individual mycotoxins depends on the climate of a given area. Some mycotoxins seem to be created in reaction to environmental change, typically due to the introduction of stress conditions, providing the producer with a competitive advantage [2]. About 400 compounds classified as mycotoxins were identified, but only a dozen were recognized as a real threat to human and animal health. Studies show that up to 60–80% of cereals may be contaminated with mycotoxins, which indicates chronic exposure of both animals and humans to mycotoxins [3]. Mycotoxin poisonings are called mycotoxicoses, and they are divided into two categories: acute and chronic. Chronic toxicity is characterized by low-dose exposure over an extended period of time, leading to malignancies and other largely irreversible effects such as cancer or immune deficiency. In contrast, acute toxicity typically has a rapid beginning and an obvious toxic response, for example vomiting [4]. In addition, mycotoxins also generate substantial monetary losses.

Mycotoxins are also often identified in cereals used to produce animal food and in ready-made mixtures. In the ten-year survey, 88% of the feed samples tested were contaminated with at least one mycotoxin, showing how unavoidable the problem of mycotoxins is, even in countries where good storage and agricultural practices are followed [5]. Such high contamination of animal feed with mycotoxins considerably threatens animal health. Mycotoxins can be immunotoxic and have a negative impact on the reproductive capacity of livestock. They can also cause histopathological changes in tissues, including kidneys, liver, and intestines, hindering the growth and survival of animals [6]. The potential for some mycotoxins to leave residues in animal products such as milk, eggs, and meat makes this issue much more significant. The residue increase the danger that mycotoxins pose to human health and the financial burden of disposing of contaminated food products [7].

Significant impact on animal health results in economic losses. Aflatoxin (AF) poisoning of crops costs Africa approximately $750 million yearly, while the EU’s regulation of AF is estimated to cost food exporters $670 million annually [8]. In the US, to manage mycotoxin-producing fungus to achieve the proper level of security, conservative estimates of expected lost crop profits and the expense of research and monitoring efforts range from $500 million to $1.5 billion per year [9].

One of the most common mycotoxins is DON, and in the previously mentioned 10-year survey, it was detected in 64% of tested feed and feed raw materials [5]. DON is a naturally occurring mycotoxin produced by certain *Fusarium* fungi; chemically, DON belongs to the trichothecenes family [10]. Due to its frequent occurrence in cereals, it is challenging to obtain feed that does not contain this mycotoxin, which makes it a great threat to agriculture even though it is not the most toxic of the known mycotoxins. In the EU, maximum levels of DON in feed have not been determined, however, guidance values have been established to help monitor and assess risk. Guidance values ​​relative to feedingstuff recommended by the European Union are 5 mg DON/kg feed. The exceptions are calves (< 4 months), lambs, kids and dogs for which the value is 2 mg DON/kg feed and pigs for which the value is 0.9 mg DON/kg feed (Table 1) [11]. Pigs are particularly vulnerable to DON due to their monogastric digestive structure and large cereal section in their diet. It is, therefore, essential to be aware of the potential adverse effects of acute or chronic DON poisoning in pigs.

**Table 1:** Commission recommendation on the presence of Deoxynivalenol in products intended for animal feeding *Fusarium* fungus infection and the presence of DON in the feed [11]

Products intended for animal feed	Guidance value in mg/kg (ppm) relative to a feedingstuff
Feed materials - Cereals and cereal products with the exception of maize by-product	8
Feed materials - maize by-product	12
Compound feed with the exception of compound feed for pigs compound feed for calves (< 4 months), lambs, kids and dogs	5
Compound feed for pigs	0.9
Compound feed for calves (< 4 months), lambs, kids and dogs	2

As was mentioned before, DON is produced by *Fusarium spp*. mainly *F. graminearum* and *F. Culmorum*. Both *F. graminearum* and *F. columorum* are plant pathogens that can cause disease in small grain crops. Research shows that DON is not produced by them under drought stress, as is the case with AF, but is associated with weather. Increased humidity caused by high rainfall correlates with a more frequent occurrence of DON, especially when increased rainfall occurs during plants’ flowering and grain development [12]. The sudden increase in DON confirmed this, observed in samples from Central and Southern Europe in 2014, where there was an increase in rainfall in July and August [5]. DON can also be made post-harvest if wet grain is not dried properly and quickly. Water activity is a physicochemical concept, that effectively quantifies the relationship between moisture in food and the ability of microorganisms to grow in them [13]. For DON to develop further, water activity (a_w_) must exceed 0.9 [12, 14]. The growing conditions of cereals are also important in preventing *Fusarium* infections. Pathogen survival is increased with reduced tillage, while conventional tillage reduces pathogens’ survival. In the fight against fungi, fungicides are also used, but their effectiveness in the fight against pathogens does not provide 100% certainty of the absence of fungi in crops. The timing and application of these products play a significant role in their effectiveness. In some cases, using fungicides may not bring the desired effects but may increase the production of DON by the fungi. It mainly occurs in suboptimal fungal growth conditions and low fungicide doses [15].

The difficulty in fighting cereal infection by *Fusarium spp*. fungi can be seen in the amount of contaminated animal feed. The formulation of feed for pigs must hit the right level of nutrients and provide an adequate supply of energy to meet the dietary requirements necessary for the healthy growth of pigs. The primary energy source for pigs is cereals, the most popular of which are wheat, barley, corn and soybean [16]. Unfortunately, these grains are often contaminated with DON, as indicated by ten year’s survey, which examined feed samples from around the world. In cereals such as wheat, barley or maize and their derivatives, more than 60% of the samples were contaminated with DON, and the values exceeded even 80,000 micrograms/kg with a median from all samples around 388 µg/kg feed. The amount of contaminated finished feed samples was also of concern, as 70% of the tested samples contained DON. It is also worth noting the number of regions where DON was the most common mycotoxin. DON was the most common mycotoxin in 8 out of 15 isolated regions. It can, therefore, be concluded that pigs are highly exposed to DON, which may be associated with numerous health consequences for animals [5].

This review aims to provide an overview of the exposure and risk of DON to pigs. It also highlights often overlooked aspects of the risk, such as the occurrence of modified forms of DON or co-occurrence with other mycotoxins, but it also shows known methods of decontamination of grains from DON.

## Bioavailability and pharmacokinetics of DON

Knowledge of the kinetics and metabolism of this compound is fundamental to assessing the risk associated with the presence of DON in pig feed. The duration of the animal’s exposure to this substance seems important in the absorption of DON. Significantly better absorption was found in pigs fed chronically with DON than in acute ones. In the study, the bioavailability of DON in chronically fed pigs was approximately 89.4%, while in acutely exposed pigs, the bioavailability was approximately 54.1% [17]. This may also be evidenced by the higher average maximum concentration achieved in the chronically exposed group of pigs than in the acute group. DON is absorbed into the pigs’ body rapidly after administration, because it was detected in the pigs’ serum just 15 min after consuming contaminated feed. Quick detection may indicate that its absorption begins in the stomach or upper part of the duodenum. Higher half-life (t1/2) and lower serum clearance were observed in chronically exposed pigs’ cases. DON is absorbed relatively quickly because it reaches its maximum concentration after a maximum of 2 h. It is also crucial that DON in serum was not detected after 24 h for the majority of tested animals [17]. The main pathways of DON metabolism are phase II metabolism and intestinal microbial transformation. In pigs, most of DON (70–94%) is glucuronidated to DON-3-glucuronide (DON-3GlcA) and DON-15-glucuronide (DON-15GlcA) in the liver and in this form, it is removed in urine in approximately 40–50%, while the rest of DON excreted in urine is mainly unchanged free DON. In a study in which urinary and serum biomarkers of DON exposure were determined, both DON and its metabolite de-epoxy-DON were undetectable in both urine and serum after less than 48 h [18]. In the digestive tract, DON, as a result of bacterial transformation, can be transformed into compounds such as de-epoxy-DON [19]. These forms show no toxic effect on the intestine, liver or lymphoid organs, as opposed to unchanged DON [20]. However, when it comes to the occurrence of this metabolite in the animal’s digestive tract, from the small intestine to the rectum, the amount of de-epoxy-DON increases progressively by up to 80% of the sum of DON and de-epoxy-DON [21]. It means that, in reality, the metabolism of DON to its less toxic metabolite in pigs is of little importance because most of DON is absorbed in the initial sections of the digestive system. This is also confirmed by the significantly higher excretion of de-epoxy-DON in faeces than in urine [21]. Research also indicates that the absorption of DON is not influenced by what part of the contaminated plant the animal eats, nor by the amount of fiber in the food, but only by the concentration of the mycotoxin contained in it [22].

## Modified forms of DON

A significant aspect of DON toxicity is its modified forms. These modified forms of mycotoxin can be produced by fungi or generated as part of the defence mechanism of the infected plant [23]. The primary derivatives of DON are acetylated forms produced by fungi, which are 3- and 15-acetyl-DON (3-ADON and 15-ADON) and modified form produced by plants - DON-3-β-D-glucoside [24].

In a study in which 82 feed samples from European countries were analysed, DON was detected in 63% and its average concentration in these samples was 948.6 µg/kg. These samples were also analysed for acetylated forms of DON and 3-ADON was found in 43% of them, while 15-ADON was present in 38%. It is worth emphasizing, however, that the concentrations of acetylate forms were much lower than DON, because they were 35.8 µg/kg and 118.3 µg/kg for 3-ADON and 15-ADON, respectively [25]. In a study of 99 feedstuff samples, DON was detected in 85% with a mean concentration of 511 µg/kg, while its acetylated forms were detected in 35% and 26% for 3-ADON and 15-ADON, respectively. DON-3-glucoside was also detected in as many as 86% of these samples, with a mean concentration of 94 µg/kg and the ratio of DON-3-glucoside/DON averaged 19% [26]. In cereal samples from Austria, Germany and Slovakia, DON and D-3-glucoside were detected in all 77 tested samples [27]. However, in the case of studies on various cereal-based products from Czech Republic, DON-3-glucoside was presented in more samples than DON, since DON was found in 76% of samples and DON-3-glucoside in 80% of them [28]. In durum wheat samples from Argentina, DON-3-glucoside was detected in 94% of samples [29]. In corn and wheat flour samples from Egypt, DON-3-glucoside was found in 32.7% of the samples and it contributed up to 33.3% of the total DON content in these samples [30]. In wheat samples from Poland, DON-3-glucoside was detected in 27% of the tested samples, with an average concentration of 41.9 µg/kg [31]. Based on literature data, European Food Safety Authority (EFSA) states that the means of the ratios calculated from the mean concentrations are as follows: 3-ADON to DON was between 0.01 and 0.49, for 15-ADON to DON from 0.01 to 0.25 and for DON-3-glucoside ranged from 0.09 is 1.49 [32].

Acetylated forms of DON are completely hydrolysed presystemically in pig’s digestive system and absorbed as DON, which is confirmed by the absence of these forms in the pig’s blood after oral administration [24, 33]. Studies using DON-3-glucoside showed it was also completely cleaved into DON before absorption. It was confirmed by studies examining the metabolism of DON-3-glucoside, after both i.v. and oral administration. When administered i.v., no hydrolysis from DON-3-glucoside to DON was observed, and DON-3-glucoside was excreted in unmetabolized form. After oral administration, DON-3-glucoside was hydrolysed and absorbed as DON, which was then metabolised to DON-glucuronide [34].

The toxic effect of acetylated forms of DON is similar to that of DON. The studies showed negative effects of 3-ADON and 15-ADON on cell production, intestinal structure and tight junctions in the pig intestine, of which 15-ADON had a more harmful effect than DON or 3-ADON [24]. The tested modified mycotoxins also caused the activation of MAPKs (mitogen-activated protein kinases), which are responsible for several cellular functions, including the expression of claudins [35]. It is also worth noting that studies have also demonstrated synergistic interactions between 15-ADON and DON [36]. Unlike DON and its acetylated forms, DON-3-glucoside in studies on porcine intestinal explants did not alter gene expression or induce histological and functional alteration [37]. However, it poses a threat due to its reconversion to DON in the gastrointestinal tract. In other studies on IPEC-J2 cell lines, the cytotoxic effect on porcine epithelial cells of these forms was ranked: DON-3-glucoside < 3-ADON < DON = 15-ADON [38].

This information emphasizes that, in addition to analysing DON in feed, attention should be paid to its modified forms due to their toxic effect or their ability to convert to DON.

## Co-occurrence of DON and other mycotoxins

Research also shows that it often we encounter not one, but two or more mycotoxins in cereals. According to the above-mentioned BIOMIN studies, more than one mycotoxin was observed in 64% of the tested samples [5]. Among those analysed by Smith et al. papers, the most common co-occurrence of DON in cereals was DON + zearalenone(ZEA), because it occurred in 14 out of 91 sources analysed. It may be due to the fact that both mycotoxins are produced by fungi from the same species, *Fusarium spp*. Other combinations observed were DON + nivalenol (NIV), DON + T2 and DON + fumonisins B1 (FB1) [39]. Studies on pig intestinal epithelial cell lines show that combinations of some mycotoxins with DON synergically increase their toxic effect. In a study by Wan et al. on IPEC-J2 cell lines in cytotoxic doses reduced cell viability significantly more in the combination of DON with NIV, ZEA, FB1, ZEA + NIV, NIV + FB1 and ZEA + NIV + FB1 than in the case of the action of one mycotoxin. Importantly, the synergistic effect of some mycotoxin combinations was observed when non-cytotoxic concentrations were used. At such concentrations, an increase in reduction in cell viability was observed in the case of the combination of DON with NIV, ZEA, NIV + ZEA and NIV + ZEA + FB1 [40]. Other studies conducted by Alassane-Kpembi et al. also observed a synergistic effect of DON and NIV on the IPEC-1 lines. However, in this study, an antagonist effect was also observed, and it occurred between DON and fusarenon-X (FX), an acetylated form of NIV. The antagonistic effect increased with the combination index between DON and FX. The authors of this publication suspect that because both compounds are substrates for the same efflux transporters, which under co-occurrence, would cause these compounds to compete for the binding site. The lower affinity of the less toxic substance may cause its cellular accumulation, leading to an overall lower toxicity of the mixture than anticipated for the combined effect [36].

## The effect of DON on the pig health

### The effect of DON on the digestive system

#### *In vivo* studies

One of the most studied side effects of DON is its negative effect on the digestive system of pigs. The first changes are usually pig’s reduced good intake and reduced weight gain [41, 42]. It may be caused by increased secretion of anorexigenic hormones such as peptide YY (PYY) and cholecystokinin. Peptide YY regulates appetite and energy homeostasis, while cholecystokinin inhibits gastric emptying and diminishes food intake [43]. The functioning of the digestive system, and therefore weight growth, is also influenced by the ability of DON to damage the cells that build the digestive tract, especially intestinal cells. DON causes changes such as shortened villi in the duodenum, but more publications describe significant changes in the jejunum of a pig’s small intestine [44]. In the jejunum, the following changes were observed: a decrease in villi height, villi pleomorphic and hyperaemia, a significant reduction in crypt depth, lesions and a decrease in the number of goblet cells [44, 45]. DON also affects the structure of the ileum, where changes similar to those in the jejunum can be noticed, i.e. lesions, reduction in crypt depth, and decrease in the number of goblet cells [45]. The reduced height of the villa is probably due to the impairment in cell proliferation, which is visible in the decrease of mitosis per microscope fields. The impairment in cell proliferation was visible in the examination of the jejunum and ileum, and this may be caused by reduced protein synthesis, which is visible in organs such as the kidneys, spleen and ileum [45–47]. The impairment of protein synthesis is also visible in reduced serum albumin concentration in animals exposed to DON and reduced expression of proteins (junction proteins), such as E-cadtherine, occludin, or claudins [41, 46]. The exact mechanism of their disturbed expression is not precisely known, but research has shown that DON, through activation of phosphoinositide 3-kinase/AKT (protein kinase B) and mitogen-activated protein kinase (MAPK) signalling pathways, can cause changes in the expression of proteins such as claudin − 4 [48]. Their reduced expression may lead to loss of enterocyte adhesive properties, leading to increased intestinal translocation of toxic luminal antigens and increased susceptibility to enteric infections [46]. DON also affects the expression of the vimentin-like gene, where vimentin is a protein expressed in the intestinal sub-epithelial layer and plays supportive roles, like promoting epithelial regrowth and enhancing barrier function during stress or inflammatory response [48]. Disturbed intestinal barrier function may also lead to disturbances in digestion and absorption of nutrients, which is visible, for example, in less apparent ileal digestibility of AA, specifically Lys, Thr, Trp and Val in growing pigs fed DON [49]. In piglets fed with feed contaminated with DON at a concentration of 1000 µg/kg, the mycotoxin caused a decrease in the thickness of the smooth muscle layer and smooth muscle cells contractile marker expression (myosin heavy chain11, smooth muscle actin gamma 2, transgelin, caponin 1) in jejunum and ileum of piglets [50]. DON also induces changes in the large intestine. In the descending colony, similarly to the small intestine, a decrease in the number of goblet cells in animals receiving a DON-contaminated diet was observed [51]. A reduced number of goblet cells is associated with a disorder of intestinal protection because mucin production is reduced [46]. A layer of mucus, known as mucins, is present throughout the gastrointestinal tract. It is made up of highly glycosylated proteins and is crucial for lubricating food passage, taking part in cell signaling pathways, and shielding the host epithelium from pathogens, commensal microorganisms, toxins, and other environmental irritants [52]. Bracarense et al. believe this may be related to the fact that DON in mucus-producing cells induces endoplasmic reticulum stress, which may lead to changes in intestinal cell density [46]. Reduced mucine production in DON-fed pigs may also be caused by the effect of DON on the protein kinase R and the mitogen-activated protein kinasep38, leading to reduced expression of resistin-like molecule β(RELM- β), which unregulates *Muc2* transcription and secretion of mucine [53, 54]. DON also leads to changes in the bacterial flora of the pig’s digestive system, causing a much greater dominance of *Lactobacillus* and *Bacteroides* species in the digestive tract in pigs fed DON compared to pigs fed with control feed. Studies have also shown changes in the stomachs of pigs exposed to DON [55]. DON at a concentration of 3.8 mg/kg caused changes in the stomach lining, or more precisely, a decrease in the depth of the gastric pits [56]. More discolouration of mucosa of the cardiac area of the stomach and epithelial thickening and keratinization of the oesophagal area of the stomach were also observed, but only in some of the research. The difference in results can be caused by different concentrations in the DON, between two tests. In research where epithelial thickening and keratinization of the oesophagal area of the stomach occurred, feed contained around 7.6 mg DON/kg feed, nearly twice as much as in other research where a smaller amount of DON in feed was present (4 mg/kg) [57, 58]. An organ that is also highly exposed to the effects of DON due to its metabolic pathway is the liver, where dose-dependent lesions occurred in animals fed with DON-contaminated feed. Changes such as vaculoral degeneration of the hepatocytes and necrosis of the individual hepatic cells were also observed [44].

#### *In vitro* studies

Many changes were also noticed in studies using cell lines derived from the pig digestive tract. In the porcine interstinal cell line (IPEC-J2), even at a concentration of 2.5 microM DON caused a decrease in cell count, and at a concentration of 10 microM cell damage, rounding of cells and autolysis were observed. DON also causes a reduction in ATP production that can impact the formation and regulation of endocytosis [59]. In studies on IPEC-J2 cell lines, DON caused the opening of mitochondrial permeability transition pores and destroyed mitochondrial membrane potential [60]. DON also caused upregulation of protein and mRNA expression of mitochondrial fission factors - Drp1, Fis1, MIEF1 and MFF (which are responsible for dividing one mitochondrion into two mitochondria) and mitophagy factors - PINK1, Parkin and LC3 (which control autophagy of mitochondria) [61, 62]. DON also downregulated mitochondrial fusion factors - Mfn1, Mfn2, except OPA1, which are responsible for the control of joining individual mitochondria [61]. These actions cause an imbalance in mitochondrial dynamics and mitophagy [60]. In studies on IPEC-J2 lines, DON also showed an effect on tight junction proteins. DON accelerated degradation of tight junction proteins in lysosome cells. The degradation of claudin-1 is influenced by the action of DON on the activation of p38 MAPK signalling pathway, while the endocytosis of claudin-1 and ZO-1 is caused by the effect of DON on c-Jun-terminal kinase (JNK). It is also confirmed by the fact that pretreatment of cells with p38 and JNK inhibitors partially restored the barrier disruption induced by DON [63]. In the Diesing et al. study the effect of DON on intestinal porcine cell lines IPEC-1 and IPEC-J2 revealed a biophasic cellular response. At higher concentrations (2000 ng/ml), DON caused disintegration of tight junction protein ZO-1, an increase of cell cycle phase G2/M, lower proliferation and cell viability and activated caspase 3, which is cell death protease and catalyse the specific cleavage of many cellular proteins [64, 65]. On the other hand, small concentrations (200 ng/ml) of DON surprisingly caused an increase in BrdU incorporation, which means a higher proliferation of cells and temporally caused Neutral red uptake reduction, which means decreased cell viability [64]. In studies using porcine enteric smooth muscle cell line, using DON at a concentration of 1000 ng/ml depresses contractility by PIMSC proliferation, migration and contractile marker expression. DON significantly downregulated MYH11, ACTG2, TAGLN, CNN1 and increased gene expression levels of MYLK and CLAM2. DON also caused the delay in wound closure in PIMSC [50].

### The effect of DON on the immune system

DON also has various effects on the functioning of the immune system in the pig body. The effect of DON was visible, for example, in the case of the disease associated with porcine reproductive and respiratory syndrome virus (PRRSV) in pigs and the effectiveness of vaccination against this disease in animals exposed to DON. PRRSV is an enveloped, positive-sense single-stranded RNA virus belonging to the Arteriviridae family. It is associated with symptoms in pigs such as acute outbreak of reproductive failure in sows, including anorexia, abortions, early farrowing, increased stillborns, mummies, weak born pigs, and delayed return to estrus, respiratory distress, fever, interstitial pneumonia, and increased preweaning mortality was present in neonatal pigs [66]. Animals exposed to feed contaminated with DON showed stronger symptoms of the disease in the form of a higher lung lesion score. In vaccinated animals, it was noted that in the case of vaccines containing attenuated viruses, DON caused a decrease in PRRSV viremia, which means that these pigs did not develop PRRSV-specific antibodies, making vaccines less effective [67]. Also, vaccinated pigs receiving feed containing 2 mg/kg DON showed clinical signs of infection as severe as unvaccinated animals, and they also displayed lower antibody titres [68]. In vaccinated animals, DON reduced the number of IFNγ producing lymphocytes. A feed contaminated with 1.09 ppm of DON caused a significantly increased frequency of TNFα^+^IFNγ^+^ producing CD4^+^T cells in the lung tissue of vaccinated pigs. This phenomenon did not occur in pigs receiving feed containing 2.81 ppm DON and receiving feed that did not contain DON. These results allow us to conclude that DON negatively affects the production of PRRSV-specific antibodies and poses a real threat of vaccine failure due to the ineffective immune response or deterioration the efficacy of vaccination against clinical signs of PRRSV [69]. The effect of DON on porcine circovirus type 2 was also examined, where although a slight increase in viral replication of this virus was observed, the difference was not statistically significant [70].

In addition to studies focusing on the direct reaction of DON to pathogens, the general effect of DON on the function of the immune system and its individual components was investigated. After two weeks of exposure to pigs, DON (2.2–2.5 mg DON/kg feed) increased the total IgA plasmatic level, while it did not increase the IgG level. A significant increase in the production of ovalbumin is also essential after immunization with ovalbumin-specific IgA, which was much more pronounced than the total IgA increase. The authors also observed the biphasic effect of DON on lymphocyte proliferation, which means that in early time points of DON exposure, there was an increase in proliferation, and later, there was a decrease after 35 days. A significant reduction of mRNA expression encoding IFNγ and TGF-β in mesenteric lymph nodes was also observed in these pigs. IFNγ and TGF-β are responsible for antimicrobial immunity and inhibition of proliferation of t-lymphocytes, respectively [71]. These changes once again confirm that DON may affect vaccinal immunity and may lead to the occurrence of disease even in properly vaccinated animals. On the other hand, other studies reported utterly different effects of DON. In this study, the level of IgA did not change due to exposure to DON, but levels of IgG and IgM significantly decreased. Studies indicate this might be happening because elevated DON concentrations lower serum IgG levels by stimulating T and B lymphocyte apoptosis and also by inducing apoptosis of B lymphocytes, decreasing IgM levels [72]. The difference between the previously mentioned study and these results may be caused by the difference in DON concentrations because in this study, a very high dose of DON was used, equal to 8 mg DON/kg feed. The presence of mononuclear lymphocytes in the kidney indicated sporadic intestinal nephritis. In kidney, a decrease in the expression of IFNγ and chemokines that play a role in the host defence against intracellular infections and a decrease in the expression of innate immune response genes, such as TNF-α and IL-6, was also noticed [72].

The impact of DON on the functioning of the immune system in various organs can be observed, for example, based on changes in gene expression in individual organs. In the liver, this mycotoxin caused the upregulation of 99 differentially expressed genes (DEGs) and the downregulation of 150 DEGs and caused the downregulation of the majority of DEGs associated with inflammatory cytokines, proliferation and other immune response networks [73]. A similar effect of DON was also observed in pig kidneys, as it caused upregulation of 120 DEGs and downregulation of 66. Here, a decrease in the expression of genes such as IL10RB, CXCL9, CXCL10 AND CCL4, which are potent inflammatory markers, was observed [74]. These results indicate the risk of suppression of the inflammatory response in the organs of pigs exposed to DON, which may lead to impaired immune homeostasis or, in the case of infection, more severe organ damage.

A significant impact of DON was also visible in the functioning of the immune system in the digestive tract of pigs. In one study, it was observed that DON at low doses (1.2–2 mg/kg) showed significant downregulation of cytokines IL-1β and IL-8 in the ileum and in blood in the same experiment, down-regulation of IL-1β, IL- 8 and TNF-α [75]. On the other hand, in another study where pigs received feed containing 2.8 mg DON/kg feed, DON significantly induced the expression of IL-1β, IL-2, IL-6, IL-12p40 and MIP-1β in the jejunum and induced the expression of TNF-α, IL-1β and IL-6 in the ileum [46]. Other studies have also demonstrated an increase in the expression of inflammatory chemokines IL8 and CXCL10, which shows that DON can induce an inflammatory response and activate Th1 response. In the jejunum of pigs fed with DON-contaminated feed, an increase in NOS2, which is an oxidative enzyme involved in generating reactive oxygen and nitrogen species by neutrophils, was also observed. In the ileum, NOS2 was not significantly up-regulated, but increased expression of GPX2, responsible for encoding the enzyme glutathione peroxidase 2 was observed. Enzyme glutathione peroxidase 2 acts as a barrier against absorption of ingested hydroperoxides and prevents intestinal inflammation, suggesting that DON may induce oxidative and inflammatory conditions in the epithelium of the ileum section. On the other hand, in this part of the digestive system, down-regulation of encoding genes encoding enzymatic antioxidants - GPX4, which enzyme protects cells against membrane lipid peroxidation, was also surprisingly observed. There was also a decrease in the expression of GPX3, which encodes an enzyme that is an extracellular antioxidant and a decrease in the expression of SOD3. SOD3 is the gene encoding superoxide dismutase 3, which is an antioxidant enzyme that catalyzes the dismutation of superoxide into oxygen [48]. This suggests that DON probably did not induce oxidative stress in the ileal lumen of pigs. In another experiment, DON caused a decrease in Total antioxidant content in the serum and an increase in urine [72]. The effect of DON on oxidative stress was also studied in pig spleen lymphocytes, where it caused the accumulation of reactive oxygen species, causing oxidative stress and increased expression of cellular mitochondrial autophagy marker proteins LC3 and P62 at the gene level. This mycotoxin also reduced expression of OPA1, mitofusion protein-1 and mitofusion protein-2, inhibiting mitochondrial fusion and promoting mitochondrial autophagy. However, it also affected mitofusion protein-2 ability to bind to the microtubule system, blocking mitochondrial autophagy transport and negatively affecting cells [76, 77]. DON also causes impaired expression of Host defence peptides (HDPs), produced by intestinal epithelial cells and responsible for effective anti-infection barrier and early response to microbial infection, inflammation and tissue injury. DON significantly downregulated intestinal HDPs expression both in weaned piglets and in vitro in IPEC-J2 cells. DON also increased caspase − 12 protein abundance, which regulates mucosal immune response and leads to a reduction in β-defensin 3 protein in the jejunum and lower NOD2 expression in the ileum, cecum and colon, which leads to lower HDPs expression in pigs fed DON-contaminated feed [78].

The effect of DON administered by intravenous injection was also tested, and in this case, it caused temporary leukocytosis related to the increase of neutrophils, which was probably associated with the elevation of serum IL-8. Increased serum concentration of IL-6 and TNF-α was also observed, which led to a significant increase in haptoglobin and serum amyloid A concentrations after 24 h from injection. The researchers also noticed increased bactericidal function of neutrophils in these pigs, which could be induced by proinflammatory cytokines including TNF-α or IL-8 [79].

The in-vitro effect of DON on pig cells was also examined. The effect of DON on polymorphonuclear cells (PMNs), which are the first line of defence against intruding microorganisms, was investigated. In vitro exposure of porcine PMNs to 10–50 µM DON decreased their chemotaxis toward IL-8, which led to impaired mobilization and recruitment of these leukocytes during infection. It also reduced the phagocytic capacity of PMNs, but did not alter the H_2_O_2_ content of the cells. DON also inhibits IL-8 secretion in LPS-stimulated PMNs and induces apoptosis in them via a process involving the permeabilization of the mitochondrial membrane, the activation of caspase-3 and the translocation of the cell membrane, which enables macrophages to recognize apoptotic cells by binding to their phosphatidylserine. This mycotoxin also induced phosphorylation of p38 [80]. In the case of peripheral blood mononuclear cells PBMC), DON at a concentration of 100 ng/Ml caused lower proliferation to mitogen-stimulated cells, but the same amount increased spontaneous proliferation. The author hypothesizes that this is caused by a possible block of the signalling from T-cell antigen receptors, where on its own, DON stimulates proliferation, perhaps activating ERK kinase. In these cells, DON in higher concentration and short-term exposure increased IL-1β and IL-8 expression, but lower concentration did not affect IL-1β and IL-8,while it decreased expression of IL-2, IL-17 IFN -γ and TNF-α and after exposure longer than five days, it decreased all the mentioned cytokines [81].

Based on these results, it can be concluded that DON definitely has immunomodulatory abilities. However, its effect on the immune system differs with the cell types, cytokine studied, and concentrations of the DON used in the research. We must not ignore the risk associated with the potential immunosuppressive effect of DON, which may lead to the development of more serious infections in pigs.

### The effect of DON on the reproductive system

Although the main systems exposed to the strong effects of DON are the digestive and immune systems, studies have also observed changes in the porcine reproductive system. The negative effects of DON affect, among others, pig oocytes. Studies have shown that this mycotoxin is able to significantly decrease the proportion of oocytes reaching metaphase II and thus disturb oocyte maturation [82]. The study also showed that in oocytes exposed to DON, this mycotoxin disrupted spindle formation, and microtubules exhibited a fuzzy appearance instead of forming normal spindle. DON also caused only a few oocytes to cleaved and no blastocyst were formed and a significant increase in the percentage of oocyte exhibiting nuclear abberations was visible [83]. Also, only 20% of oocytes treated with DON underwent parthenogenetic activation, which means that DON affected meiotic cell cycle progression. Oocytes exposed to DON also showed enhanced expression of LC3 protein and autophagic-related genes Lamp2, LC3 and reduced expression of mTOR. These changes demonstrate early apoptosis in DON-treated porcine oocytes, which led to a reduction in the quality of oocytes. An important effect of DON may also be its ability to perform epigenetic modification, such as DNA methylation, which was increased in DON-treated oocytes by altered DNMT3 mRNA level. DNA methylation is essential for later embryo development during oogenesis, which means that DON might affect oocyte maturation via DNA methylation. DON also changed levels of protein like H3K27me3, H3K4me2 and H3K9me3. These changes are significant for oocyte maturation because H3K27me3 is essential in embryonic genome activation, H3K4me2 is a crucial regulator during early development and is a hallmark of transcriptional activation and H3K9me3 is involved in transcriptional silencing, are associated with oocyte developmental competence [84]. In the case of porcine uterine cells, exposure to DON significantly decreased cell number and caused cells changes, such as swollen mitochondria, disrupted cell membranes and many vacuoles. It also had anti-proliferative effects by controlling the progression of cells through the cycle by decreasing S-phase and arresting cells in the Go/G1 phase of the cell cycle. There was also a visible decrease in the expression of the monitored proliferating marker, which was the proliferating cell nuclear antigen (PCNA). This decrease in the expression means that DON can disengage cells from active cycling [85]. In a study of pig’s ovaries explants, it was observed that DON increased the lesion score and degeneration of the oocytes and granulosa cells, interstitial oedema and pyknotic cells. It also decreased the number of normal follicles by increasing degeneration type II in primordial, primary and growing follicles [86].

As for the effect of DON on the cells of the male reproductive system, DON in combination with ZEA showed oxidative damage to the sertoli cells, which are the somatic cells of testis, essential for spermatogenesis. Mycotoxins disrupt their cell cycle, destroy tight junction proteins and promote cell apoptosis via the mitochondrial pathway [87]. In studies on boar, DON also influenced total sperm, progressive motility (agglutination and asthenospermia) and sperm chromatin structure [88]. In contrast, in another study DON did not affect DNA integrity, but had a negative effect on two important computer-assisted semen analysis parameters, which were immotile and progressive motile spermatozoa. It also negatively affected sperm morphology and viability [89].

Regarding the fetus and the impact of DON on its development, in piglets exposed intrauterine to DON, DON concentration in piglets blood was the highest at 12 h after delivery and decreased during the first week of life. What is surprising, however, is that DON was detectable in the plasma of the piglets up to 14 weeks, which is very different to older pigs, where DON is completely eliminated within 24 h. This phenomenon can be explained by physiologically lower liver capacity in young piglets and lower activity capacity of the hepatic enzymes involved in DON metabolism. They also had a decrease in immunoglobulins in the first week of life and significant differences in the percentages of T cell subset: CD4 + Th cells, and CD8hi Tc cells. γδ T cells and T-reg, where the most decreased groups were CD8 positive CD + Th cells and T-regs, which are important in maintaining of immune response and T cell homeostasis [90].

DON also influenced the capacity to produce T cell-related cytokines IFN-γ, IL-17, IL-2 and TNF-α, where this mycotoxin caused their reduced expression and in the case of some of these cytokines this effect remains even at 18 weeks after birth, where DON was not detectable in the plasma [90]. It is also important that DON has the ability to pass through the placenta barrier, which also exposes fetuses whose mothers consume feed contaminated with DON. The study observed that DON reduced the number of monocytes and neutrophils and increased lymphocytes in fetuses whose mothers consumed contaminated feed [91]. In studies combining DON with ZEA, these mycotoxins caused reduced maternal and tended to reduce the weight of piglets of sow eating contaminated diet from day 75 to 110 of pregnancy [92]. Fetuses whose mothers were fed with these mycotoxins also had increased glycogen content and changes in the architecture of mitochondria in their livers, which may have resulted from diaplacentar toxin transfer [93].

## DON decontamination

Due to the high occurrence of DON in feed, methods of decontaminating grains from this mycotoxin are currently being sought. Currently known methods can be divided into physical, chemical and biological [94, 95].

Physical methods include those that use materials capable of adsorbing DON. Such materials include silicate minerals, activated carbon, nano zeolite and synthetic resin [96]. In an in vitro model that uses two different buffers to simulate gastrointestinal conditions, in pH 3.5 the highest DON adsorption was observed in Calcium lignosulphonate (87%) and in activated charcoal (70%). Unfortunately, in pH 7, authors observed desorption of DON reaching 100% from Calcium lignosulphonate and 59% from activated charcoal [97]. In other recent studies, adsorption of DON was observed in 4–16% by Cross-linked chitosan, 35% by microsphere adsorbent containing an alginate/carboxymethyl cellulose sodium composite loaded with calcium (SA/CMC-Ca) and 37% by ion-exchanged zeolites [98–100]. Montmorillonite is a compound that arouses interest and is widely researched in the context of DON decontamination. Montmorillonite is a mineral clay, that is a member of the smectite group [101]. This clay showed adsorption potential and the results were even 3–4 times better when pillar montmorillonite was used instead of normal montmorillonite, since the adsorption was up to 35% [102]. Unfortunately, this mineral works in a low acidic environment, which can influence the original nutrition of the feed [102]. A physical way to degrade DON may also be through heat treatment. Unfortunately, however, in the case of DON, this method is not highly effective due to its high heat resistance, stability at temperatures up to 120 degrees Celsius, and moderate stability at temperatures up to 180 degrees Celsius [103]. The use of a temperature high enough for effective DON degradation is associated with loss of nutrients in the feed and may change its taste [104, 105]. Physical methods of DON degradation that do not involve high temperatures include UV, irradiation or atmospheric cold plasma. The use of UV-C treatment caused a significant reduction in DON concentration. However, this method is highly dependent on feed matrix or exposure time, and generally, light does not penetrate solid food such as grains, which makes this method less practical [106, 107]. Also, it is inefficient and can cause changes in the taste and nutrient composition of feed. Gamma radiation is one of the most effective radiation methods for decontaminating DON. However, it is mainly effective for dissolved DON, and when used on dried maize, DON was resistant to degradation [108, 109]. The mechanism of action of gamma radiation on DON probably causes this difference. In the presence of water, gamma radiation causes free radicals formation, which may react with this mycotoxin. Another limitation of this method is that large irradiation doses must be used to be effective [109]. A promising method is atmospheric cold plasma, which caused almost complete DON degradation after 60 s [110]. This method uses plasma, a form of ionized gas that has a high concentration of charged particles, reactive chemicals, excited molecules, and UV photons, the combined effect of which, contributes to highly effective DON degradation [111]. However, more research is needed to understand the mechanism of action of this method thoroughly.

When it comes to chemical methods, they use bases and acids or gases such as ozone. Various sodium compounds have been successful in the chemical degradation of DON. The use of compounds such as Na2S2O5 or Na2O3 solution on DON caused a decrease in the DON concentration through its degradation or the formation of DON-sulfonate, which in studies does not show toxic effects [112, 113]. Sodium metabisulfite also demonstrated the ability to degrade DON, which caused a significant decrease in DON concentration in acidic aqueous conditions [114, 115]. These methods, however, have little practical application because toxin removal efficiency is low, the treatment cost is high, and they can destroy many nutrients in grains. The gas currently used in the food industry is ozone. Ozone removed 26% of the DON found in infected wheat. The effectiveness of this method increases with the increase in ozone concentration, exposure time and with increasing moisture. DON in High-moisture wheat was less resistant to ozone exposure than DON in low-moisture wheat in the same ozone exposure conditions. Despite the method’s effectiveness, it is unsuitable for large-scale use due to costs and the need for special equipment [116].

The next group are biological methods, which are based on the adsorption of mycotoxins into microbial cell walls and the degradation of DON by enzymes produced by microorganisms. The walls of some bacteria contain compounds that can induce adsorption mechanisms, such as hydrogen bonds or ions interactions. Microorganisms interacting with DON include yeast, lactic acid bacteria or mycelia, and some filaments fungi [117–119]. Research in which DON was suspended in PBS buffer, *Lactobacillus* strains were able to remove DON by an average of 30%. However, for the more expensive strains *Saccharomyces cerevisiae*, the mean decrease was 33% in 24 h [117]. When effect of *Saccharomyces pastorianus* on a wort containing mycotoxins was examined, DON was removed by an average of 15%, and DON-3-Glucoside by 17% [120]. In the studies using spiked with mycotoxins, beer fermentation residue containing *Saccharomyces cerevisiae*, DON adsorption was observed at a level of 11.6% at pH and DON adsorption at a level of 17.6% at pH 6.5 [121]. Regarding the enzymatic degradation of DON by bacteria, the most important pathways are (1) oxidation of DON to 3-keto-DON [122] and, (2) destroying the epoxy structure of DON, degrading it into DOM-1 [123]. Both of these compounds are less toxic than DON. The advantages of these methods are their high specificity, efficiency, and lack of toxic secondary products.

A 100% effective method of feed decontamination with DON that would not affect the feed’s nutrient content and would be feasible to use on a large scale has not yet been found.

## Conclusion

DON is one of the most common mycotoxins in the world and is present in up to 60% of the feed samples tested, which shows how great a threat it is to animals that eat feed containing mainly cereals. One such animal is the pig, which, due to its diet and poor DON metabolism, is particularly sensitive to the potentially toxic effects of this mycotoxin.

This review collects information about the kinetics of DON in the pig body and its metabolism. DON is absorbed into the pigs’ body relatively quickly, and then metabolised mainly in the liver. The main route of DON excretion is the urinary system, where both unchanged and metabolized forms are removed in the urine. Modified forms of DON have also been described, which may arise under the influence of fungi or plants. They pose a threat not only because of their toxic effect on the epithelium of the small intestine of pigs, but mainly because both the acetylated forms and DON-3-glucoside are converted to DON in the digestive tract. In the case of co-occurrence, attention was also paid to the possibility of DON interactions with other mycotoxins. The literature contains information on the occurrence of DON, mainly with other mycotoxins also produced by fungi of the *Fusarium spp*. species. Unfortunately, studies have also observed synergistic effects between some of them, the exact mechanisms and potential interactions that may occur in the animal’s body have not yet been thoroughly investigated. DON affects many systems in the pig’s body, but this review collects information on the effects of DON on systems where the most pronounced effects of DON toxicity are visible. The most affected by the harmful effects of DON are the digestive track, immune and reproductive systems. In the digestive track, the most observed changes under the influence of DON were visible in the small intestine of pigs. However, they can also be seen to a lesser extent, in the large intestine and liver, which are also exposed to the action of this mycotoxin due to its metabolism. As for the immune system, DON has shown immunosuppressive effects in many studies, which may negatively affect the course of infection in animals exposed to this mycotoxin. In the reproductive system, DON disturbs the proper development of cells, which is important in the reproductive process. It may lead to impaired reproduction in pigs and decreased fertility.

However, some of the effects of DON are not completely consistent between different studies, which may be due to different mycotoxin concentrations, different exposure times, and different experimental conditions. Nevertheless, it is obvious that DON has a negative impact on pigs’ bodies and poses a real threat to their health, thereforeit should be controlled in feed intended for animals. It is especially important because a 100% effective method of feed decontamination with DON that would not affect the feed’s nutrient content and would be feasible to use on a large scale has not yet been found.

## Data Availability

No datasets were generated or analysed during the current study.

## Abbreviations

ZEA: Zearalenone
DON: Deoxynivalenol
DON-3GlcA: DON-3-glucuronide
DON-15GlcA-DON: 15-glucuronide
3-ADON: 3-acetyl-deoxynivalenol
15-ADON: 15-acetyl-deoxynivalenol
AF: Aflatoxin
MAPK: Mitogen-activated protein kinase
NIV: Nivalenol
FB1: Fumonisins B1
FX: Fusarenon-X
PYY: Peptide YY
RELM-β: Resistin-like moleculeβ
IPEC-J2: Intestinal porcine epithelial cells
JNK-c: Jun-terminal kinase
PRRSV: Porcine reproductive and respiratory syndrome virus
DEG: Differentially expressed gen
HDP: Host defence peptides
PMN: Polymorphonuclear cells
PBMC: Peripheral blood mononuclear cells
PCNA: Proliferating cell nuclear antigen
